# Identification of Candidate Biomarkers in Malignant Ascites from Patients with Hepatocellular Carcinoma by iTRAQ-Based Quantitative Proteomic Analysis

**DOI:** 10.1155/2018/5484976

**Published:** 2018-09-23

**Authors:** Jinyan Zhang, Rong Liang, Jiazhang Wei, Jiaxiang Ye, Qian He, ChunlingYuan, Jiazhou Ye, Yongqiang Li, Zhihui Liu, Yan Lin

**Affiliations:** ^1^Department of Medical Oncology, Affiliated Cancer Hospital of Guangxi Medical University, Nanning 530021, China; ^2^Department of Otolaryngology & Head and Neck, The People's Hospital of Guangxi Zhuang Autonomous Region, Nanning 530021, China; ^3^Institute for the Advancement of Higher Education, Hokkaido University, Sapporo 060-0817, Japan; ^4^Department of Hepatobiliary Surgery Oncology, Affiliated Cancer Hospital of Guangxi Medical University, Nanning 530021, China

## Abstract

Almost all the patients with hepatocellular carcinoma (HCC) at advanced stage experience pathological changes of chronic liver cirrhosis, which generally leads to moderate ascites. Recognition of novel biomarkers in malignant ascites could be favorable for establishing a diagnosis for the HCC patients with ascites, and even predicting prognosis, such as risk of distant metastasis. To distinguish the proteomic profiles of malignant ascites in HCC patients from those with nonmalignant liver cirrhosis, an iTRAQ pipeline was built up to analyze the differentially distributed proteins in the malignant ascites from HCC patients (n=10) and benign ascites from hepatic decompensation (HD) controls (n=9). In total, 112 differentially distributed proteins were identified, of which 69 proteins were upregulated and 43 proteins were downregulated (ratio <0.667 or >1.3, respectively) in the malignant ascites. Moreover, 19 upregulated proteins (including keratin 1 protein and rheumatoid factor RF-IP20, ratio>1.5) and 8 downregulated proteins (including carbonic anhydrase 1, ratio<0.667) were identified from malignant ascites samples. Functional categories analyses indicated that membrane proteins, ion regulation, and amino acid metabolism are implicated in the formation of HCC malignant ascites. Pathways mapping revealed that glycolysis/gluconeogenesis and complement/coagulation cascades are the mostly affected cell life activities in HCC malignant ascites, suggesting the key factors in these pathways such as Enolase-1 and fibrinogen are potential ascitic fluid based biomarkers for diagnosis and prognosis for HCC.

## 1. Introduction

Hepatocellular carcinoma (HCC) is the fifth most prevalent cancer in the world and a leading cause of cancer-related death worldwide [1]. Age-adjusted incidence rates among HCC patients have increased faster in recent decades while its mortality is almost equal to its morbidity. Development of HCC is asymptomatic at early stages of the disease, which is attributed to complex causes including chronic liver diseases, hepatitis virus infection, and alcohol abuse. HCC patients rarely can be diagnosed at early stage due to lack of effective biomarkers for the diagnosis. A few blood test based biomarkers like alpha fetal protein (AFP) are used in clinical practice, but the value of these tests for surveillance purposes has not been sufficiently validated. There is an urgent clinical need to identify new HCC biomarkers for improvement of diagnosis and treatment outcome monitoring [2].

Ascites is the pathologic accumulation of fluid in the peritoneal cavity that exceeds certain amount. A majority of HCC are observed in association with liver cirrhosis, while ascites is one of the major complications of liver cirrhosis and indicates poor prognosis [3]. HCC patients generally have malignant ascites which is bloody and opaque compared to benign ascites which is mostly clear. The cell growth of primary tumor and its metastasis in other parts of body contribute to the formation of malignant ascites, suggesting molecular analysis of malignant ascites from cancer patients may provide valuable information for clinical surveillance, medical screening, and intervention [4]. Ascitic fluid has been analyzed in order to develop a differential diagnosis for malignancy-related cirrhosis, including liver cirrhosis. For instance, one recent study reported that lactoferrin level in ascites is a useful diagnostic tool to identify in cirrhosis patients with spontaneous bacterial peritonitis [5]. Studies of ascitic samples on oncology research were largely focused on breast cancer, and gastric cancer and colorectal cancer, but rarely in HCC [6–8]. Despite the large amount of information reported about the characterization of ascitic fluid, its diagnostic value in HCC patients is not yet defined.

Other than the widely used genomic analyses such as microarrays and NGS sequencing technologies in molecular biomarker discovery, proteomic-based approaches have shown many advantages in protein biomarkers screening [9–11]. Moreover, protein based studies can assist in systems biology research which could lead to identification of new drug targets [12]. Isobaric tag for relative and absolute quantitation (iTRAQ) is an approach using a multiplexed set of four or eight isobaric reagents to tag protein/peptide and then measure their relative expression levels [13]. This technique allows the protein samples to be pooled after labeling without increasing the complexity of measurement, making it feasible to identify and quantify proteins simultaneously. Compared with other proteomic-based techniques, iTRAQ has been employed to proteomics research on various types of samples including cell lines, tissues, fluids, and even bacteria [14–16]. Hundreds of unique proteins identified by iTRAQ have been predictive in the translational research and clinical practice [17, 18].

In the presented study, an iTRAQ pipeline was built up to analyze the differentially distributed proteins in HCC ascetics samples when compared with hepatic decompensation (HD) controls. In total, 19 upregulated proteins and 8 downregulated proteins were identified from ascitic fluid samples (HCC* vs.* HD). Identified upregulated proteins included keratin 1 and rheumatoid factor RF-IP20 while the downregulated proteins included carbonic anhydrase 1. Functional categories analyses showed membrane proteins are key factors in the development of HCC malignant ascites. Pathways mapping revealed pathways of glycolysis/gluconeogenesis and complement/coagulation cascades are the mostly affected functions involved in HCC malignant ascites, suggesting key factors from these pathways could serve as biomarkers for HCC.

## 2. Materials and Methods

### 2.1. Patient Subjects

Patients with primary hepatocellular carcinoma (HCC, n=10) and hepatic decompensation (HD, n=9) were enrolled and their ascetic fluid samples were collected. Characteristics of the enrolled patients are shown (Supplementary Materials, Table S1). The diagnosis for the HCC patients was according to the histological results of liver surgical resections. All protocols related human materials were approved by the ethics committee of Affiliated Cancer Hospital of Guangxi Medical University. Informed consent was obtained and the access to human samples was carried out in accordance with the approved guidelines of the ethics committee.

### 2.2. Ascitic Fluid Collection

Malignant fluid samples were collected from patients by paracentesis done under sterile condition using 21-gauge needle. The routine testing of ascitic fluid included cell counting and measurement of total protein and albumin. The specific investigations like liver biopsy and ascitic fluid culture were performed as required. Cell debris in the ascetic fluid was removed through centrifugation at 4°C for 20 min at 15,000*g*. The supernatant was transferred to a sterile tube and the amount of total protein was determined by using a commercial assay reagent kit (Pierce BCA Protein Assay Kit). For the preparation of samples used in iTRAQ assay, 100 *μ*g of each sample was collected carefully in cold sterile PBS containing protease inhibitor cocktail and stored at −70°C immediately. For the subsequent ELISA assay, the total amount of proteins from 10 mL of each ascitic fluid sample was harvested and stored as described above. No additional freeze-thaw was carried out before the detection.

### 2.3. iTRAQ Proteomics Analysis

The mixed ascitic fluid samples were submitted for iTRAQ analysis (Beijing BangFei Bioscience). Each sample was labeled using iTRAQ Reagent-8plex Multiplex Kit according to the protocol (Applied Biosystems, Foster City, CA). Eight isobaric tags were employed to label the samples from HCC group or HD group. The labeling strategy was established according to the concentration and distribution of total amount protein from all samples measured by BCA protein quantification. The iTRAQ labeling protocol is illustrated (Supplementary Materials, Figure S1). Sample fractionation was carried out by using SCX chromatography column (C18, 3 *μ*m, 0.1 × 2.0 cm polysulfoethyl A column, PolyLC Inc.), as described previously [19]. LC buffers were prepared freshly every day as follows: (1) SCX chromatography buffer A (SCX-A): 20% (vol/vol) acetonitrile and 0.1% (vol/vol) formic acid (pH 2.7); (2) SCX chromatography buffer B (SCX-B): 20% (vol/vol) acetonitrile, 0.1% (vol/vol) formic acid and 1 M KCl (pH 2.7). In brief, 100 *μ*l of SCX-A was added to each sample and pooled to a single tube; each tube was further rinsed with 100 *μ*l of SCX-A and added to pooled material. Each entire sample was transferred to a glass sample vial and the volume was adjusted to 1.6 ml with SCX-A and loaded onto the analytical column for a HPLC system (Agilent, Palo Alto, CA) using a 2-ml injection loop and washed with SCX-A at 1 ml min^−1^ for 40 min. To acquire the separated component, a binary mobile-phase gradient at a total flow rate of 250 *μ*l mL/min was applied. The gradient comprised an increase from 0 to 14% SCX-B over 24 min, 14 to 30% SCX-B over 36 min, and 30 to 100% SCX-B in 20 min. The column was then washed with 100% SCX-B for 15 min and reequilibrated with 100% SCX-A for 15 min.

### 2.4. LC-MS/MS Analysis

The amount of 10 *μ*L loading buffer was added to each sample in a high PH condition to dissolve the labeled samples before running with Q Exactive HF Orbitrap LC-MS/MS System (Thermo Finnigan). All samples (each 2.5 *μ*g) were analyzed using an Easy nLCsystem HPLC coupled to a Q Exactive mass spectrometer. Peptides were preconcentrated on a C18 trapping column (3mm × 10 mm × 20 mm, PolyLC Inc.) for 10 min using 0.1% TFA (v/v) with a flow rate of 250 *μ*L/min followed by separation. The Q Exactive HF MS was operated in data-dependent acquisition (DDA) mode and MS survey scans were acquired from m/z 300 to 1,400 at a resolution of 120,000. Isolation of precursors was performed by the quadrupole with a window of 1.6 m/z. The most intense signals were subjected to higher energy collisional dissociation with a normalized collision energy (0.054*∗*m/z + 5) taking into account a dynamic exclusion of 12.0s. Maximum injection times (IT) were 45 ms. Precursor ions with charge states of +1, +7, +8, or >8 or unassigned were excluded from MS/MS analysis.

### 2.5. Protein Quantification and ELISA

The MS/MS data were searched against the Mascot database (uniprot-human_20151227.fasta) for peptide identification and quantification. The search result of peptide was filtered by FDR* p* value with a cutoff of 0.01. The differentially distributed proteins were further characterized using the software Proteome Discoverer 1.4 (Thermo). To further verify the validity of the iTRAQ-based quantitative proteomic analysis, an enzyme-linked immunosorbent assay (ELISA) was carried out to examine and quantify level of carbonic anhydrase I (CA1) within the ascitic fluid samples, which was detected to be decreased in the HCC group. The ready-to-use ELISA Kits were purchased from LifeSpan BioSciences Inc. (catalog No. LS-F23971) and the experiment was performed in accordance with the manufacturer's protocol.

### 2.6. Statistics Analysis

Statistical analysis was performed using SPSS Statistics 22.0 (IBM). Differences analyses in protein expression between the HCC and HD groups were performed using a* t*-test, and p < 0.05 was taken to indicate statistical significance. Based on statistical dispersion of the dataset (a total of 627 proteins were detected), ratio of >1.3 or <0.767 was set as the threshold to identify differently expressed proteins, using a cutoff of two times standard deviation (Supplementary Materials, Figure S2). In addition to that, ratio of >1.5 or <0.667 was used as a strict significance cutoff to acquire a short list of the differentially distributed proteins as indicated in the data legends. Eukaryotic Orthologous Groups (KOG) and Gene Ontology (GO) and Kyoto Encyclopedia of Genes and Genomes (KEGG) analyses were considered statistically significant at p < 0.05.

## 3. Results

### 3.1. Differentially Distributed Proteins Identified by iTRAQ Analysis

**Figure 1:**
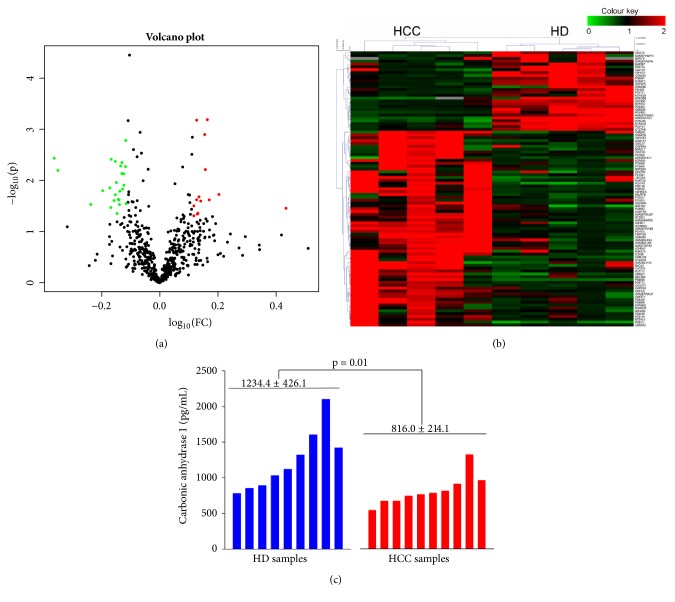
**Differentially distributed proteins identified by iTRAQ quantitative analysis. (a)** Volcano plots showing differentially distributed proteins comparing HCC and HD. Each point represents the difference of fold-change (X axis, Log_10_[fold change]) plotted against the level of statistical significance (Y axis, Log_10_[*p* value]). Proteins represented by red dots indicate upregulation and green dots indicate downregulated proteins (p<0.05, ratio >1.3 or < 0.767).** (b)** Hierarchical clustering of the differentially distributed proteins identified from each samples (ratio >1.3 or < 0.767). The color scale indicates the expression level of each protein across the two groups.** (c) **ELISA assay for carbonic anhydrase I (CA1) in the ascitic fluid samples.

To identify HCC-specific protein biomarkers from HCC ascites proteome, the ascitic fluid samples from HCC patients and HD control were collected and analyzed by iTRAQ and MS analysis. Out of the total 627 proteins detected in the assay, 112 differentially distributed proteins were identified, of which 69 proteins were upregulated and 43 proteins were downregulated (ratio <0.767 or >1.3) (Supplementary Materials, Table S2). With Proteome Discover 1.4 software, the distribution of significantly changed proteins detected in ascites samples was illustrated in a volcano plot (*t*-test p<0.05, ratio >1.3 or < 0.767, Figure 1(a)) and the expression levels of all proteins in each sample were visualized in a hierarchical clustering heatmap (ratio >1.3 or < 0.767, Figure 1(b)). The green dots in Figure 1(a) and cluster A in Figure 1(b) indicate the downregulated proteins in HCC* vs.* HD while the red dots and cluster B indicate the upregulated proteins. With more strict criteria for significance (*t*-test p<0.05, ratio >1.5 or < 0.667), a total of 27 proteins were identified as the differentially distributed proteins in HCC* vs.* HD comparison, including 19 upregulated proteins and 8 downregulated proteins. The top ranked differentially distributed proteins are listed in Table 1. To further validate the effectiveness of the iTRAQ analysis, ELISA assay was employed to determine the concentrations of carbonic anhydrase I (CA1) in the ascitic fluid samples. The results indicated that the level of CA1 in HCC was significantly lower than that in HD group (Figure 1(c)), which was consistent with the results from iTRAQ analysis (Table 1).

### 3.2. KOG and GO Classifications

**Figure 2:**
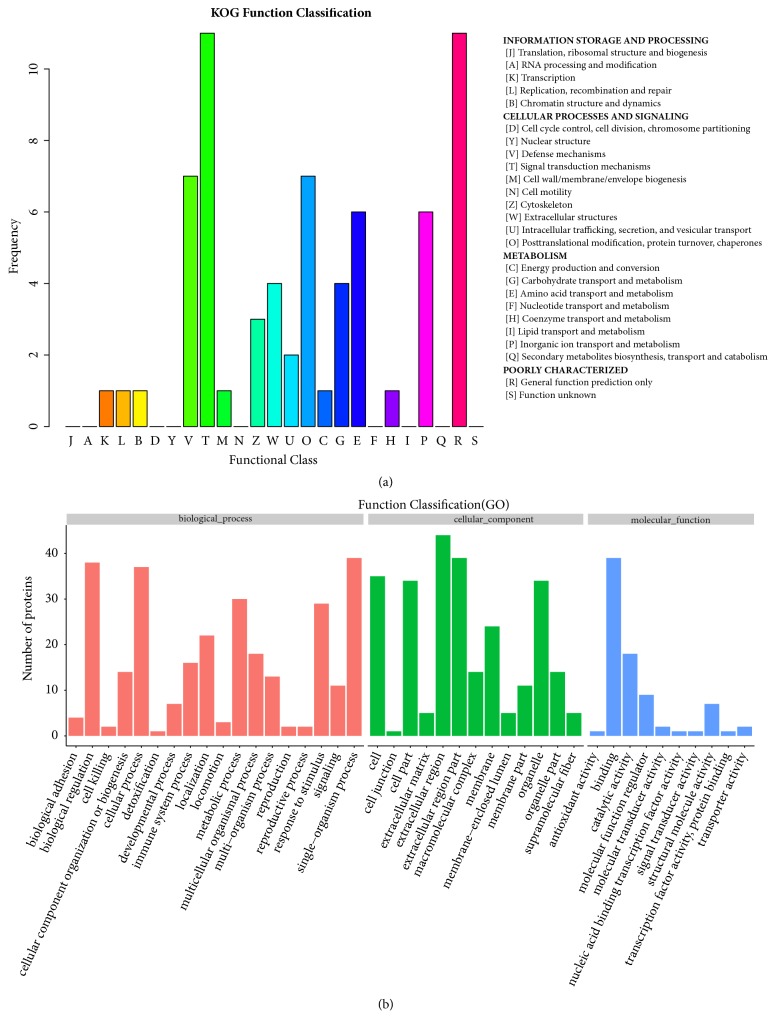
**COG functional categories and GO classification. (a)** Functional assignments of the differentially distributed proteins to the KOG (Eukaryotic Orthologous Groups) categories. The main KOG categories are illustrated with different colors and the frequency of each category is shown.** (b)** Overall GO (Gene Ontology) analysis of all differentially distributed proteins (HCC* vs.* HD) in accordance with the biological processes (left), cellular component (middle), and molecular function (right). Protein numbers in each category are shown.

In the following studies, all 110 proteins identified in the above data were classified using KOG and GO analyses to predict their possible roles in malignant ascites. The KOG database is a phylogenetic classification of the gene products from completely sequenced genomes [20]. Annotated proteins in KOG are assumed to originate from ancestor proteins, which reflect the system evolution relationships of the individual proteins. In our KOG functional classification, all the differentially distributed proteins from HCC* vs.* HD were annotated into 16 KOG categories (Figure 2(a)). The top ranked functional clusters included 3 largest categories in cellular processes and signaling group (signal transduction mechanisms, defense mechanisms and posttranslational modification, protein turnover, and chaperones) followed by 2 smaller categories in metabolism group (amino acid transport and metabolism, inorganic ion transport and metabolism).

GO analysis is widely used to describe molecular function of protein sets [21]. In our functional GO analysis, all of the differentially distributed proteins were mapped to terms in the GO database. The results showed the proteins belonged to 47 categories grouped into 3 major clusters including biological process, cellular components, and molecular function (Figure 2(b)). The most commonly enriched GO terms of HCC specific proteins were extracellular region and extracellular region part.

The KOG and GO analyses indicated that protein signaling transduction and extracellular region are major affected function involved in HCC malignant ascites. These data indicated that most of the differentially distributed proteins between HCC ascites and HD ascites are likely membrane proteins that are able to bind with specific ligand and activate the signaling transduction.

### 3.3. KEGG Pathway Identification

**Figure 3:**
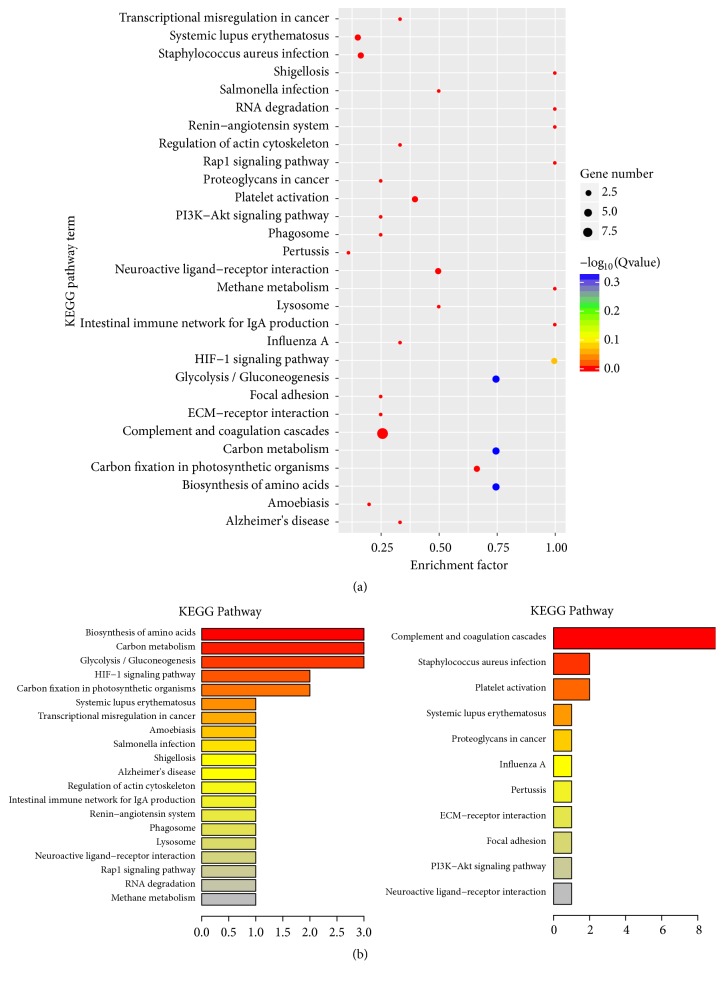
**KEGG pathways in HCC ascites samples compared to HD. (a)** The most significantly enriched KEGG (Kyoto Encyclopedia of Genes and Genomes) pathways are illustrated with bubbles. The Y axis indicated the enrichment factor, which refers to the ratio of the differentially distributed protein number to the total protein number in a certain pathway. The size of bubble indicates mean number of proteins enriched in a given pathway. The color of bubble indicates Q value (adjusted* p* value).** (b)** Bar plots represent enriched KEGG pathways associated with upregulated (left) proteins and downregulated (right) differentially distributed proteins. The Y axis indicated average change fold of the implicated proteins.

Kyoto Encyclopedia of Genes and Genomes (KEGG) is a database resource designed for characterization the high-level biological functions based on proteomic data. KEGG pathways were constructed to better understand the biological pathways and acquire the molecular mechanisms involved in HCC malignant ascites development. A summary of overall KEGG pathways (Table 2), KEGG pathways associated with upregulated (Figure 3(b), left) proteins and downregulated (Figure 3(b), right) differentially distributed proteins, was provided. KEGG pathway analysis of all the differentially distributed proteins found four enriched pathways with enrichment factor (the differentially distributed protein number/total protein number) higher than 0.75, including glycolysis/ gluconeogenesis pathway, carbon metabolism, and biosynthesis of amino acid pathway (all p<0.05). All of the 4 pathways are activated in HCC malignant ascites and associated with the upregulated proteins in HCC malignant ascites. The most inactivated pathway represented by downregulated protein is complement and coagulation cascades (p<0.10).

### 3.4. Signal Pathway Analysis

**Figure 4:**
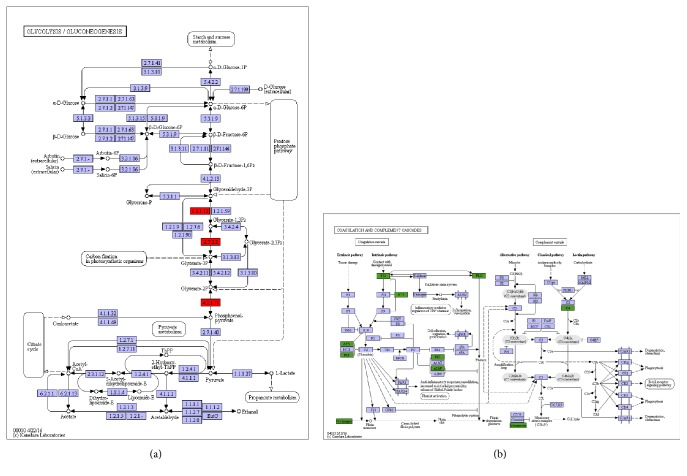
**The differentially distributed protein levels in top ranked pathways. (a)** Upregulated proteins in glycolysis/gluconeogenesis pathway. 1.2.1.12, GAPDH; ENO1 (Enolase-1); 2.7.2.3, PGK1 (phosphoglycerate kinase 1).** (b)** Downregulated proteins in coagulation and complement cascades pathway. C4, CO4B; fibrinogen, FIBA; AT3, ANT3 (antithrombin III); PLG, PLMN (plasminogen); vitronectin, VTNC; *α*2AP, A2AP; F12, coagulation factor III. The regulated proteins in HCC vs. HD are marked in colored boxes. Red box indicates an upregulated protein; green box indicates a downregulated protein; gray box indicates that no significant difference was observed.

The glycolysis/gluconeogenesis pathway was found significantly activated in HCC ascites, including significantly upregulated proteins like Enolase-1 and phosphoglycerate kinase 1. These proteins were involved in metabolism of glycolysis, which is known for a potent driving force of tumor growth and therapy failure (Figure 4(a)). Another pathway coagulation and complement cascade was also focused here due to its central function in controlling fibrin clot formation. This pathway exhibits the highest number of assigned differentially distributed proteins identified in the above data. Many coagulation products, such as fibrinogen, antithrombin, plasminogen, and vitronectin, were downregulated in HCC* vs.* HD (Figure 4(b)). These results suggested the HCC ascites specific proteins reflect a response to local tumor microenvironment and could serve as an important attribute of disease pathogenesis.

## 4. Discussion

To the best of our knowledge, here we reported for the first time the proteomic profiling of HCC ascites using iTRAQ-based proteomic profiling, which is of high prognostic importance and a valuable tool to identify biomarkers not only to distinguish the malignant HCC from benign ascites caused by other chronic liver diseases, but also to predict disease progression during ascites formation. More importantly, a few functional categories and key factors were found affected in HCC ascites, indicating the potential of these molecular signatures as biomarker for diagnosis and prognosis. In our study, 112 differentially distributed proteins (69 upregulated and 43 downregulated) were characterized from ascitic fluid samples of HCC patients, including keratin 1 (KRT1), keratin 2 (KRT2), carbonic anhydrase 1 (CA1), and hepatocyte growth factor activator (HGFA).

With the increasing complexity of HCC, scientists have to explore new biomarkers to meet the demands from clinical diagnosis. Although the molecular mechanisms by which HCC develops remain unknown due to its heterogeneity, a multitude of proteomic, pathological, and molecular signatures of HCC have been uncovered and modeled to predictive or diagnostic biomarker. iTRAQ technique has been used to discover new protein biomarkers in almost every field of clinical diagnosis and translational research. Patients with chronic liver disease can present with acute decompensation due to various causes, including benign ascites and HCC [22]. Compared to serum or plasma based biomarker, proteomic analyses of body fluid could directly evaluate the local liquid microenvironment of cancer cells, which is a major reason why we chose to identify biomarkers for HCC. Our data also provided additional proof that the iTRAQ technique is capable of quantifying the protein levels change from ascitic fluid samples.

One of the gold standards for HCC diagnosis is alpha-fetoprotein (AFP), which is widely used in China and other Asian countries [23]. Recent studies have identified some other protein biomarkers which are able to be supplementary to AFP in the detection of HCC, such as Glypican-3 (GPC-3), Osteopontin (OPN), Golgi protein-73, squamous cell carcinoma antigen (SCCA), Annexin A2, and Thioredoxins [24, 25]. Most of the current protein biomarkers for HCC were focused on blood based specimen and showed low specificity for diagnosis or progression prediction. Similar to our study, a new protein biomarker S100A9 recently was identified to be upregulated in the tumor tissue interstitial fluids (p<0.05, ratio >1.3) [26]. ROC analysis showed this protein has sensitivity of 91% (higher than AFP) and specificity of 66% when used to distinguish HCC from liver cirrhosis (LC, HCC high risk population). The proteins identified in the present study have shown strong oncogenic ability with HCC or even biomarker potential in previous studies, suggesting these profiling data fit with some current hypotheses. For instance, keratin family proteins such as keratin 19 indicate high risk of tumor metastasis, invasion, and poor prognosis in HCC patients [27, 28]. Carbonic anhydrase 1 was found to be downregulated in HCC samples by iTRAQ-based quantitative proteomic analysis, which was confirmed by ELISA (Figure 1(c)). Carbonic anhydrases function by maintaining acid-base balance in blood and other tissues and participating in transporting carbon dioxide out of tissues. Carbonic anhydrase was also found to be correlated with tumor progression and predicted poor survival of HCC patients with high tumor stage. The role of carbonic anhydrases in ascites formation progression still remains poorly understood. Characterization of the implication of carbonic anhydrases in HCC ascites might reveal underlying mechanisms through which malignant ascites were generated, and carbonic anhydrases could potentially serve as ascites based biomarkers for HCC.

Over the past decade, the importance of the tumor microenvironment in HCC progression has been recognized but has not been well defined yet. It has been demonstrated that the malignant cells and the molecular signatures of ascites are changed continuously during the course of HCC [29]. It is not surprising that KOG and GO analyses on the presented data demonstrated that several key cellular functions were affected in HCC ascites, including signal transduction mechanisms, defense mechanisms and posttranslational modification, protein turnover, and chaperones. In addition to that, key metabolic processes including amino acid transportation and inorganic ion transportation were enriched in HCC group compared to HD control group. These findings were suggestive of the underlying mechanisms that regulate tumor cell growth and cellular metabolism in malignant ascetics and may help elucidate the molecular basis of HCC progression.

The signaling pathway signatures we reported here will shed light on the biomarker discovery in ascites and other inflammatory fluid. In particular two pathways were highlighted in the current study. Enzymes such as Enolase-1 and phosphoglycerate kinase in glycolysis/gluconeogenesis pathway were found significantly activated in HCC ascites. Our data is consistent with previous reports that Enolase-1 is expressed remarkably differently between the HCC tissue samples and precancerous lesions [30]. Moreover, serum antibodies against Enolase-1 are potential biomarkers for predicting Enolase-1 in HCC prior to surgical resection [31]. These biomarkers should be further investigated as potential therapeutic targets. These results indicated that HCC ascites specific proteins such as Enolase-1 responded and altered proteostasis in the unique cellular environment of HCC malignant ascetics and may predict HCC progression or be used as a potential therapeutic target for HCC.

Our study also suggested that coagulation and complement pathway is implicated in HCC malignant ascites formation, and the coagulation products (such as fibrinogen and plasminogen) might indicate status of HCC progress. Serum fibrinogen level is positively correlated with advanced tumor stage and poor survival in gastric cancer patients, suggesting fibrinogen protein as a biomarker for predicating tumor progression and survival of the patients [32]. Agreeing with our data, increased tendency to hemorrhage was observed in cancer patients [33]. A previous study reported a scoring system using combination of fibrinogen concentration and neutrophil-lymphocyte ratio to predict tumor progression in gastric cancer [34]. Circulating fibrinogen was found to be a prognostic and predictive biomarker in malignant pleural mesothelioma [35]. The similar findings were also reported in lung cancer, cervical cancer, protest cancer, and breast cancer [36–38]. A very recent report found high fibrinogen level in plasma is significantly associated with poorer overall survival of HCC patients with or without radical therapies [39]. Consistent with that, our study showed fibrinogen level was decreased in HCC ascites compared with benign ascites, suggesting its potential to predict prognosis of the patients with HCC.

Taken together, the analysis of malignant ascites has identified numerous potential biomarkers that could provide more information underlying HCC metastasis and progression. Some potential biomarker, such as Enolase-1 in glycolysis/gluconeogenesis pathway and fibrinogen in coagulation and complement cascades pathway, as discussed above, should be further determined in additional samples to validate the results presented in this study. Further investigation in a larger study population with a rigorous selection of the candidate proteins is needed in order to validate the clinical values of the biomarkers. Efforts should be directed towards prospective clinical trials in evaluating the prognostic significance of these candidate markers.

## 5. Conclusion

Utilizing iTRAQ-based proteomics analysis, the profiles of malignant ascites from HCC patients were characterized by identifying the differentially distributed proteins compared with benign ascites. Glycolysis/gluconeogenesis and complement/coagulation cascades are remarkably affected in HCC malignant ascites, which strongly suggests the protein molecules involved in these pathways such as Enolase-1 and fibrinogen are potential ascitic fluid based biomarkers for not only establishing diagnosis but also predicting clinical outcomes for HCC.

## Figures and Tables

**Table 1:** Top upregulated and downregulated proteins identified by iTRAQ (HCC *vs*. HD, p<0.05).

**Accession**	**Description/Gene name (Protein name)**	**Ratio (HCC/HD)**	**Score**
**Upregulated proteins**			

B4E1B2	cDNA FLJ53691	1.60	4358
Q6MZU6	DKFZp686C15213	2.21	3984
A2MYE1	VACWR153(A30 protein)	1.55	493
A2J1N3	Rheumatoid factor RF-IP20	2.72	332
A8K9J7	H2B(Histone H2B)	1.97	121
H6VRF8	KRT1(Keratin-1)	1.88	110
B0AZL7	cDNAFLJ79457(highly similar to Insulin-like growth factor-binding protein)	1.66	62
A0N8J1	V(k)3 sequence of NG9 gene from fetal liver DNA	1.52	57
P35908	KRT2(Keratin-2)	1.53	51
Q04756	HGFA(Hepatocyte growth factor activator)	1.59	45

**Downregulated proteins**			

Q6PIQ7	IGL(Immunoglobulin lambda)	0.61	3129
Q6DHW4	Uncharacterized protein	0.60	526
A0A0J9YXX1	Uncharacterized protein	0.43	146
E5RIF9	CA1(Carbonic anhydrase 1)	0.57	104
Q5NV65	V1-5(V1-5 protein)	0.64	45

**Table 2:** Top upregulated and downregulated KEGG pathways identified by iTRAQ (HCC *vs.* HD).

**Pathway ID**	**Pathway Name**	**Gene Name**
**Upregulated proteins**		

ko00010	Glycolysis / Gluconeogenesis (p<0.05)	GAPDH, ENO1,PGK1
ko01200	Carbon metabolism (p<0.05)	GAPDH,ENO1,PGK1
ko01230	Biosynthesis of amino acids (p<0.05)	GAPDH,ENO1,PGK1
ko00710	Carbon fixation in photosynthetic organisms	GAPDH,PGK1
ko04066	HIF-1 signaling pathway	GAPDH,ENO1
ko00680	Methane metabolism	ENO1
ko03018	RNA degradation	ENO1
ko04015	Rap1 signaling pathway	PFN1
ko04080	Neuroactive ligand-receptor interaction	CTSG
ko04142	Lysosome	CTSG
ko04145	Phagosome	MPO
ko04614	Renin-angiotensin system	CTSG
ko04672	Intestinal immune network for IgA production	PIGR
ko04810	Regulation of actin cytoskeleton	PFN1
ko05010	Alzheimer's disease	GAPDH
ko05131	Shigellosis	PFN1
ko05132	Salmonella infection	PFN1
ko05146	Amoebiasis	CTSG
ko05202	Transcriptional misregulation in cancer	MPO
ko05322	Systemic lupus erythematosus	CTSG

**Downregulated proteins**		

ko04610	Complement and coagulation cascades (p<0.10)	CO4B, FIBA, FGB ANT3, PLMN, VTNC A2AP, IPSP, Q8IZZ5
ko04611	Platelet activation	FGA
ko05150	Staphylococcus aureus infection	C4B
ko04080	Neuroactive ligand-receptor interaction	PLG
ko04151	PI3K-Akt signaling pathway	VTNC
ko04510	Focal adhesion	VTNC
ko04512	ECM-receptor interaction	VTNC
ko05133	Pertussis	CO4B
ko05164	Influenza A	PLG
ko05205	Proteoglycans in cancer	VTNC
ko05322	Systemic lupus erythematosus	CO4B

## Data Availability

The data in an Excel file used to support the findings of this study are included within the Supplementary File S1.

## Supplementary Materials

Supplementary 1 Table S1: characteristics of the 19 enrolled patients are shown.

Supplementary 2 Supplementary Figure S1: iTRAQ labeling strategy and study protocol are illustrated.

Supplementary 3 Supplementary Figure S2: histograms above indicate the iTRAQ-based quantification ratio (HCC vs. HD) distribution. X axis: Log2 (fold-change); Y axis: frequency (sample number).

Supplementary 4 Supplementary File S1: data of quantitative proteomic analysis for a total of 627 proteins detected in this study is shown.
