# Supplementary material for: Identification of Candidate Biomarkers in Malignant Ascites from Patients with Hepatocellular Carcinoma by iTRAQ-Based Quantitative Proteomic Analysis
Source: Biomed Res Int. 2018 Sep 23;2018:5484976. doi: 10.1155/2018/5484976 (PMC6174818; doi:10.1155/2018/5484976)
Supplement: Supplementary 2 — Supplementary Figure S1: iTRAQ labeling strategy and study protocol are illustrated. [file 5484976.f2.pptx]

## Slide 1
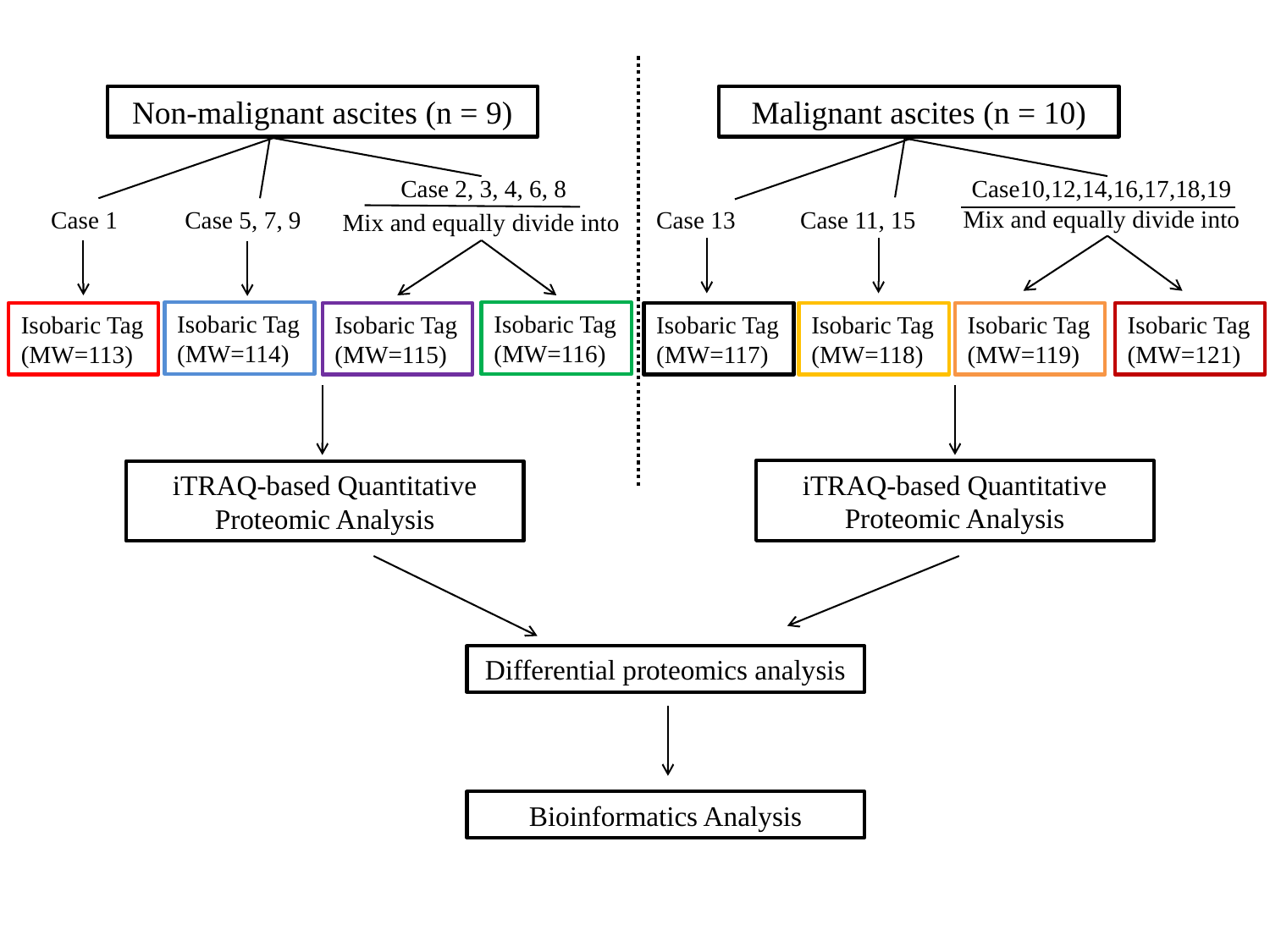

Non-malignant ascites (n = 9)
Malignant ascites (n = 10)
Case 2, 3, 4, 6, 8
Case10,12,14,16,17,18,19
Case 11, 15
Case 13
Case 1
Case 5, 7, 9
Isobaric Tag (MW=114)
Isobaric Tag (MW=116)
Isobaric Tag (MW=115)
Isobaric Tag (MW=117)
Isobaric Tag (MW=118)
Isobaric Tag (MW=119)
Isobaric Tag (MW=121)
iTRAQ-based Quantitative Proteomic Analysis
iTRAQ-based Quantitative Proteomic Analysis
Differential proteomics analysis
Bioinformatics Analysis
Mix and equally divide into
Mix and equally divide into
Isobaric Tag (MW=113)
