# Supplementary figures and images for: Identification of Candidate Biomarkers in Malignant Ascites from Patients with Hepatocellular Carcinoma by iTRAQ-Based Quantitative Proteomic Analysis
Source: Biomed Res Int. 2018 Sep 23;2018:5484976. doi: 10.1155/2018/5484976 (PMC6174818; doi:10.1155/2018/5484976)

## Slide 1
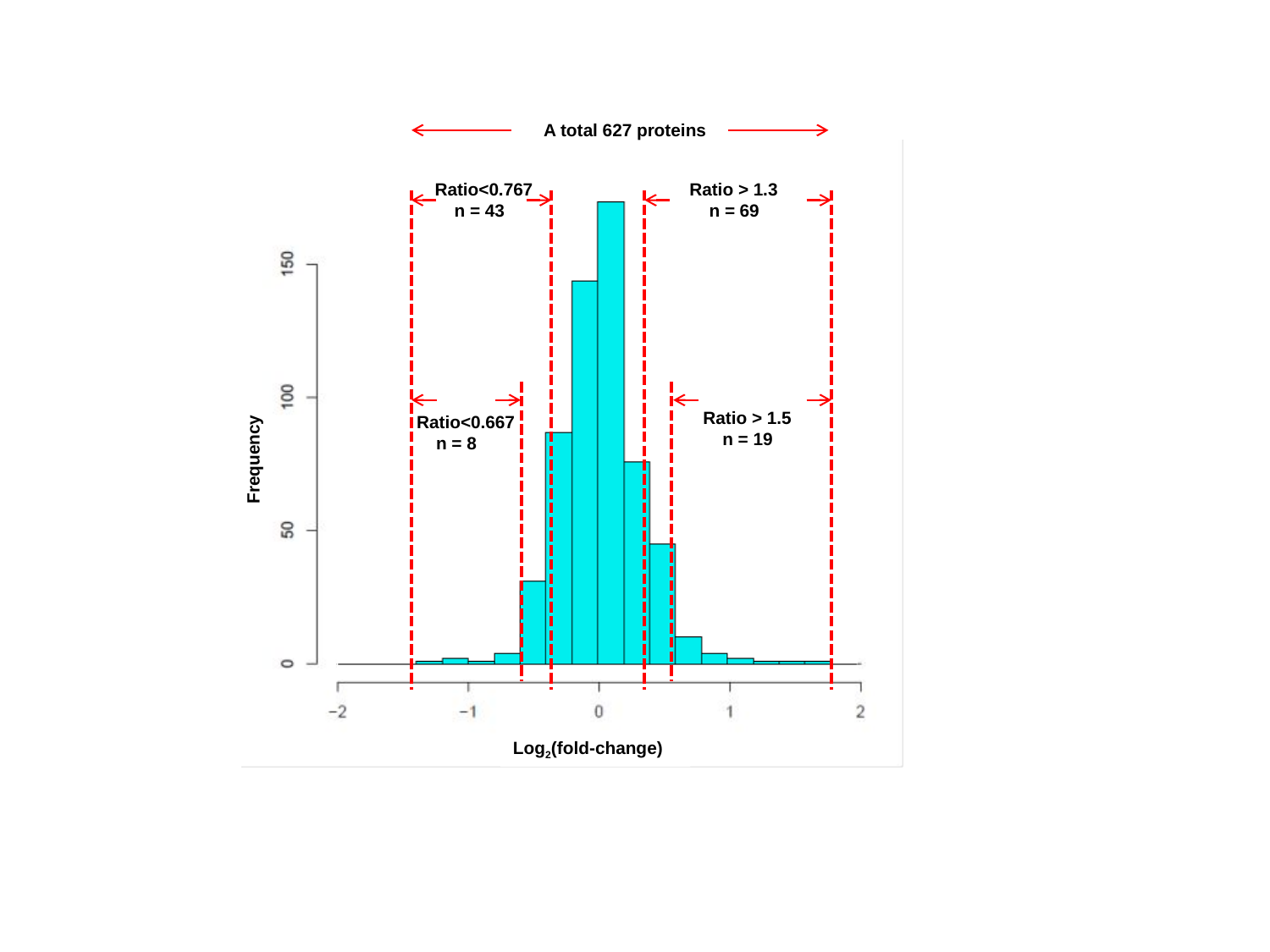

A total 627 proteins
Ratio<0.767
 n = 43
Ratio > 1.3
 n = 69
Ratio > 1.5
 n = 19
Ratio<0.667
 n = 8
 Frequency
Log2(fold-change)

Supplement: Supplementary 3 — Supplementary Figure S2: histograms above indicate the iTRAQ-based quantification ratio (HCC vs. HD) distribution. X axis: Log2 (fold-change); Y axis: frequency (sample number). [file 5484976.f3.pptx]
