# Supplementary material for: Identification of Candidate Biomarkers in Malignant Ascites from Patients with Hepatocellular Carcinoma by iTRAQ-Based Quantitative Proteomic Analysis
Source: Biomed Res Int. 2018 Sep 23;2018:5484976. doi: 10.1155/2018/5484976 (PMC6174818; doi:10.1155/2018/5484976)
Supplement: Supplementary 4 — Supplementary File S1: data of quantitative proteomic analysis for a total of 627 proteins detected in this study is shown. [file 5484976.f4.docx]

**Table S1 Characteristics of the enrolled patients**

| ID | Gender | Age | Diagnoses | Protein concentration (μg/μL) |
| --- | --- | --- | --- | --- |
| 1 | Male | 54 | Alcoholic liver cirrhosis (decompensated) | 18.1 |
| 2 | Male | 49 | Viral liver cirrhosis (decompensated) | 25.5 |
| 3 | Male | 53 | Viral liver cirrhosis (decompensated) | 17.5 |
| 4 | Male | 59 | Viral liver cirrhosis (decompensated) | 26.0 |
| 5 | Male | 50 | Viral liver cirrhosis (decompensated) | 20.5 |
| 6 | Male | 47 | Viral liver cirrhosis (decompensated) | 24.8 |
| 7 | Male | 65 | Viral liver cirrhosis (decompensated) | 21.6 |
| 8 | Male | 63 | Viral liver cirrhosis (decompensated) | 23.7 |
| 9 | Female | 64 | Viral liver cirrhosis (decompensated) | 17.8 |
| 10 | Female | 61 | Hepatocellular Carcinoma | 12.6 |
| 11 | Male | 40 | Hepatocellular Carcinoma | 11.3 |
| 12 | Male | 43 | Hepatocellular Carcinoma | 18.9 |
| 13 | Male | 48 | Hepatocellular Carcinoma | 14.9 |
| 14 | Male | 65 | Hepatocellular Carcinoma | 15.1 |
| 15 | Male | 56 | Hepatocellular Carcinoma | 9.1 |
| 16 | Male | 42 | Hepatocellular Carcinoma | 14.2 |
| 17 | Male | 47 | Hepatocellular Carcinoma | 17.4 |
| 18 | Male | 57 | Hepatocellular Carcinoma | 12.5 |
| 19 | Male | 40 | Hepatocellular Carcinoma | 12.2 |

Characteristics of the 19 enrolled patients are shown.

**Supplementary Figure S1**

**
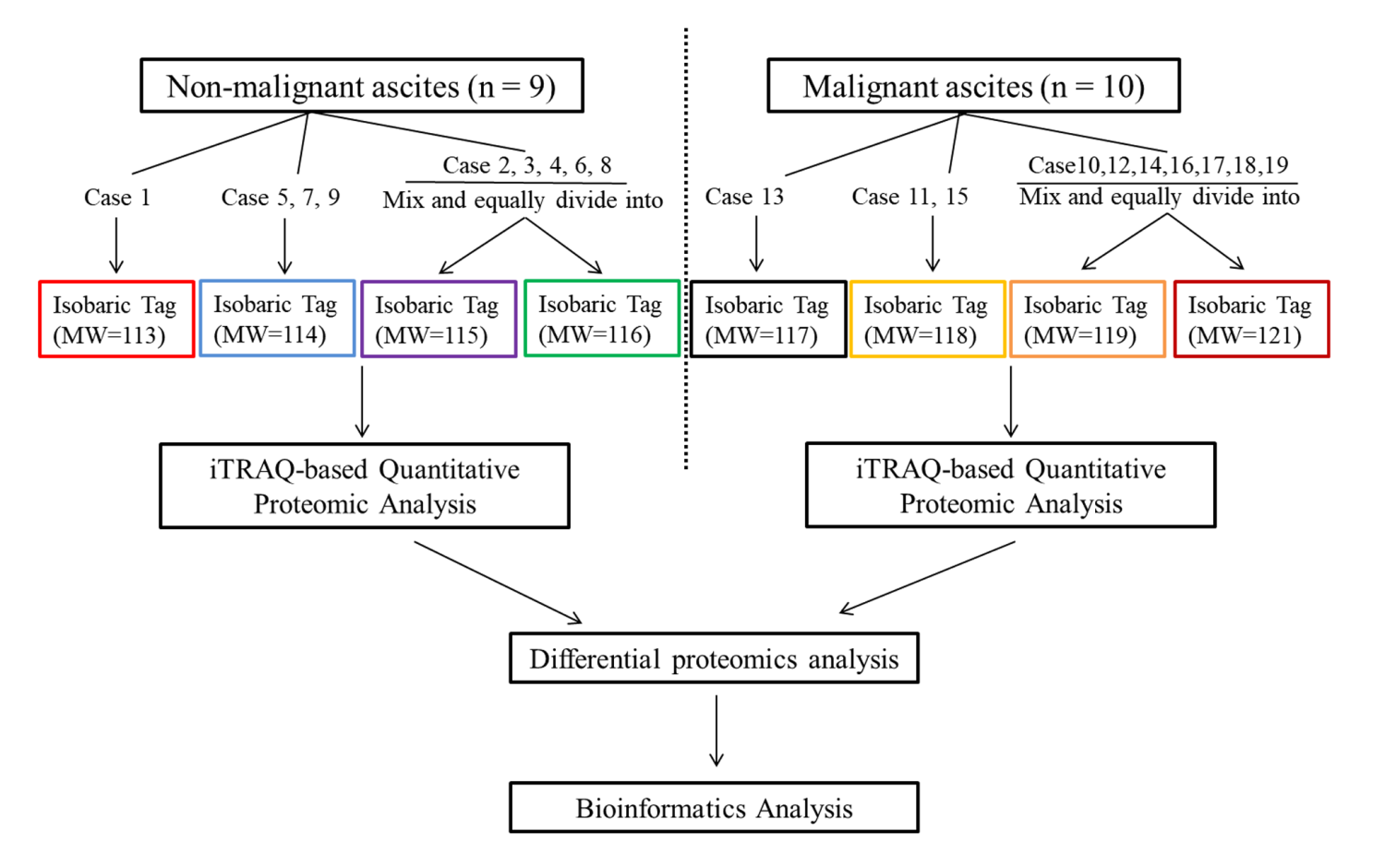
**

iTRAQ labeling strategy and study protocol are illustrated.

**Supplementary Figure S2**

**
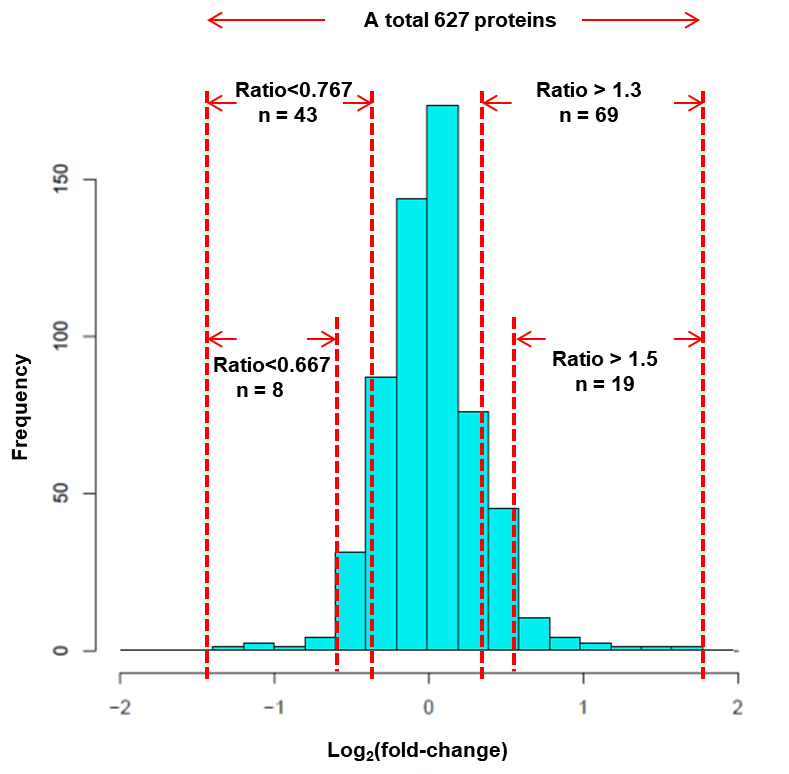
**

Histograms above indicate the iTRAQ-based quantification ratio (HCC *vs.* HD) distribution.

X axis: Log_2_(fold-change); Y axis: Frequency (sample number).

**Supplementary File S1**

Data of quantitative proteomic analysis for a total of 627 proteins detected in this study is shown.
